# A Research Ward at St. Thomas's Hospital

**Published:** 1914-06-27

**Authors:** 


### A RESEARCH WARD AT ST. THOMAS'S HOSPITAL.

A proposal is on foot at St. Thomas's Hospital that, as a new departure, a Research Ward be provided in the hospital and that some beds in this Research Ward should be definitely allotted to cases under investigation by members of the staff. Apparently it will be left with the Research Committee to arrange with a senior physician or surgeon to be responsible for the clinical care of the patients. Research Assistants would be appointed, each of whom would be paid.

This bold and far-reaching proposal requires most careful deliberation. Not only does it involve, as has been pointed out, very important principles, but it may be expected to commit the hospital to a considerable expenditure both for capital and for upkeep. As practical men of affairs, the governors are bound to keep this latter aspect of the matter in view, even if they are absolutely unanimous about the former.

The proposal for a research ward embodies an idea which has possibly been borrowed from Cambridge. There exists in that town a small hospital, "supported by voluntary contributions," the main object of which is not the relief of as many of the sick poor as possible, but the promotion of research and the provision of materials for it. This unique hospital, which has been described on a former occasion in The Hospital (June 1, 1912, page 223), was originated mainly by Dr. Pigg Strangeways, and owes an enormous debt of gratitude to him for his energy in procuring for it financial support as well as workers. At all events, the proposal laid before the St. Thomas's authorities appears to be an application of the same idea to the circumstances of a big general hospital. For the mere advancement of such a revolutionary—or rather evolutionary—change must prove stimulating to the imagination of voluntary hospital managers all over the kingdom. Those institutions which have faith enough and courage enough to lead the way, in our opinion, will assuredly reap a due reward in public confidence, in public estimation, and, what is more, in public support and munificence.

We have not heard whose is the mind that originated this proposal, but we congratulate the St. Thomas's authorities upon their enterprise in considering the new departure officially from the point of view of practical politics. Whatever be the measure of its realisation, it may prove to mark the beginning of a new development in institutional medicine and surgery.

